# Cardiovascular and diabetes burden attributable to physical inactivity in Mexico

**DOI:** 10.1186/s12933-020-01050-3

**Published:** 2020-06-29

**Authors:** Catalina Medina, Pamela Coxson, Joanne Penko, Ian Janssen, Sergio Bautista-Arredondo, Simón Barquera, Kirsten Bibbins-Domingo

**Affiliations:** 1 Department of Physical Activity and Healthy Lifestyles, Center for Nutrition and Health Research, National Institute of Public Health, Mexico City, Mexico; 2 Department of Epidemiology and Biostatistics, Center for Vulnerable Populations, University of California, San Francisco, CA USA; 3 Department of Medicine, University of California, San Francisco, CA USA; 4 School of Kinesiology and Health Studies, Queen’s University, Kingston, ON Canada; 5 Division of Health Economics and Health Systems Innovations, Center for Health Systems Research, National Institute of Public Health, Cuernavaca, Morelos Mexico; 6 Center for Nutrition and Health Research, National Institute of Public Health, Cuernavaca, Morelos Mexico; 7 Division of General Internal Medicine, Zuckerberg San Francisco General Hospital, University of California, San Francisco, PO BOX 1364, San Francisco, CA 94143-1364 USA

**Keywords:** Physical inactivity, Cardiovascular diseases, Type 2 diabetes, Mortality, Mexico

## Abstract

**Background:**

Physical inactivity (PI) is associated with the development of non-communicable chronic diseases. The purposes of this study were to estimate the extent to which the 31% relative increase in PI among 35–64 years old Mexicans between 2006 and 2012 influenced diabetes (T2D) and cardiovascular disease (CVD) incidence and mortality, and to estimate the impact of the World Health Organization recommended 10% and 15% relative decrease in PI on CVD and T2D incidence and mortality by 2025 and 2030, respectively.

**Methods:**

Estimates were derived using the Cardiovascular Disease Policy Model-Mexico, a computer simulation, Markov model. Model inputs included cross-national data on PI levels from 2006 and 2012 measured using the International Physical Activity Questionnaire and the published literature review on the independent relationship between PI and cardiometabolic risk.

**Results:**

The models estimated that the 31% increase in PI resulted in an increase in the number of cases of T2D (27,100), coronary heart disease (10,300), stroke (2200), myocardial infarction (1500), stroke deaths (400) and coronary heart disease deaths (350). A hypothetical 10% lowering of PI by 2025 compared to status quo is projected to prevent 8400 cases of T2D, 4200 cases of CHD, 1000 cases of stroke, 700 cases of MI, and 200 deaths of CHD and stroke, respectively. A 15% reduction resulted in larger decreases.

**Conclusions:**

While the burden of T2D and CVD raised from 2006 to 2012 in association with increased PI, achieving the WHO targets by 2030 could help reverse these trends.

## Background

Non-communicable diseases (NCDs) are the leading causes of death worldwide [1]. NCDs are responsible for more than 70% of deaths globally, with the majority of those occurring in low-and-middle income countries [1]. In Mexico, coronary heart disease (CHD) and type 2 diabetes (T2D) are the leading causes of death [2]. Although Mexico has implemented some cost-effective strategies to reduce this burden [3], public health policies are still needed to reduce risk factors for these diseases, including physical inactivity (PI) [4].

The World Health Organization (WHO) defines PI as not accumulating at least 150 min/week of moderate physical activity (PA) or 75 min/week of vigorous PA or an equivalent combination of the two intensities (i.e., < 600 MET-minutes per week of moderate-to-vigorous PA) [5].

In Mexico, the prevalence of PI in adults aged 20–69 year increased by 44% from 2006 to 2012 [6]. This is troubling given that PI increase the risk of CHD by 16% [7], stroke by 18% [8] and T2D by 16% [8]. The upward PI trend of in Mexico contrasts starkly with the global targets set by the WHO. Specifically, the WHO has called for a 10% relative reduction in the prevalence of PI by 2025 and 15% relative reduction by 2030 [9].

Some studies have estimated the extent to which changes in PI in the population influence NCD. [10, 11] For instance, Lobello and colleagues estimated that an absolute reduction of PI of 30% in 2002 would reduce T2D deaths by 5.3% [10] and Katzmarzyk et al. estimated that a 10% relative reduction in PI in Canada would reduce stroke deaths by 19.9% [11]. To our knowledge, similar estimates have not been generated for Mexico or other Latin American countries. Furthermore, studies have not estimated how many NCDs would be prevented in the 2025 and 2030 WHO PI targets were achieved [9].

Thus, the objectives of our study were both to estimate the extent to which the observed increase in PI in Mexico from 2006 to 2012 influenced the incidence and mortality of cardiovascular disease (CVD) and T2D, and to project the potential reductions in CVD and T2D that could be achieved if the 2025 and 2030 WHO PI targets are attained.

## Methods

### The Cardiovascular Disease Policy Model—Mexico

The estimates in this paper were generated using the *Cardiovascular Disease Policy Model (CVD Policy Model).* This model has been used for over 30 years to forecast CVD incidence, prevalence, mortality and costs among the 35 to 94-year-old US population. The US Model was adapted to represent the population of Mexico as described previously [12]. The model is a computer simulation, state transition (Markov) model composed of three sub-models. The demographic-epidemiological submodel stratifies the population without preexisting CVD into cells defined by sex, 10-year age categories, and risk factor distributions for smoking status, systolic blood pressure, HDL and LDL cholesterol, body mass index, the presence of T2D, and levels of PI. In annual cycles, the Model predicts the incidence of T2D, CHD, stroke, and non-CVD death among those without preexisting CVD using risk functions estimated from Framingham Original and Offspring cohort data [13, 14]. The bridge submodel captures the incident event (cardiac arrest, myocardial infarction, angina, or stroke) and sequelae in the 30 days following the event. The disease history submodel stratifies the population with CVD into cells defined by age, sex, and CVD history. Those with prior CVD then have annual rates of recurrent events, revascularization procedures, and cardiovascular and non-cardiovascular death with transition probabilities determined from natural history studies, hospital databases, and calibration of event rates to achieve total cardiovascular events and deaths reported in Mexican hospital and vital statistics data (Additional file 1: Appendix S1) [15, 16].

### Model inputs

The base year of the CVD Policy Model-Mexico is 2010, with model inputs derived from nationally representative data sources wherever possible (Table 1, Additional file 1: Appendix S3). We obtained data on risk factors distribution, transition between risk factors and PI from the 2006 and 2012 cross-sectional National Mexican Health and Nutrition Surveys (ENSANUT). These surveys used a probabilistic multistage stratified cluster design [17, 18]. Each ENSANUT cycle is a national representative survey of adults aged 20 years or higher who represented more than 50,000,000 people. Detailed descriptions of the ENSANUT methodology are published elsewhere [17, 18].

**Table 1:** Inputs used for the CVD Policy Model-Mexico

Parameters (year)	**Source**
Mexican population estimates (2010)	Instituto Nacional de Estadística, Geografía e Informática (INEGI) [22]
Population projection estimates (2010–2030)	Consejo Nacional de Población (CONAPO) [23]
Total and cause-specific mortality (2010)	Sistema Nacional de Información en Salud (SINAIS) [24]
CVD incidence (2010)	Instituto Mexicano del Seguro Social (IMSS) [25], Instituto de Seguridad y Servicios Sociales de los Trabajadores del Estado (ISSSTE) [26], Sistema Nacional de Información en Salud (SINAIS) [24]
CVD deaths (sudden cardiac death, arrest survival to hospital, case fatality rates, revascularization rates) (2000 and 2010)	Instituto Mexicano del Seguro Social (IMSS), Instituto de Seguridad y Servicios Sociales de los Trabajadores del Estado (ISSSTE), Sistema Nacional de Información en Salud (SINAIS) [24], USA National Hospital Discharge Survey (NHDS) [27, 28]
Risk functions for incident CVD and non-CVD deaths (2010)	Framingham Heart Study Original Cohort [13] and Offspring Cohort [14]
Transition between risk factors (2006)	Encuesta Nacional de Salud y Nutrición (ENSANUT) 2006 [29]
Physical inactivity prevalence (2006 and 2012)	Encuesta Nacional de Salud y Nutrición (ENSANUT) 2006 [29] and 2012 [6]
Risk factors distribution (2006 and 2009)	Encuesta Nacional de Salud y Nutrición (ENSANUT) 2006 [29], Encuesta Global de Tabaquismo en Adultos (GATS) 2009 [30]
Relative risk of physical activity levels on disease incidence (2011 and 2016)	Kyu et al. (T2D and stroke) [8] and Sattelmai et al. (CHD) [7]

Both, ENSANUT 2006 and 2012 used the Spanish version of the short form International Physical Activity Questionnaire (IPAQ) to estimate PI prevalences [19]. The IPAQ has been developed and tested for use in adults aged 15 to 69 year old [19]. This instrument has been validated in a Mexican population with a modest reliability (r = 0.55) and poor validity against accelerometer (r = 0.31) [20]. Based on the WHO guidelines, we classified participants as PI if they did not accumulated at least 150 min of moderate-to-vigorous physical activity (PA) per week (< 600 MET-minute per week) or physically active if they accumulated this amount of PA [5, 21]. We determined the prevalence of PI in 2006 and in 2012 among 22,995 and 6273 adults 35–64 years that had complete values representing 29,846,493 and 35,734,922 people respectively. We then stratified this prevalence by sex and 10-year age groups [6].

We obtained inputs on the association between PI and incident T2D, CHD and stroke through a literature review of meta-analyses. We used the following criteria for the selection of meta-analyses: (1) Relative risk (RRs) were estimated for studies that assessed total PA based on the accumulation of PA across four domains (leisure-time, occupational, transport and household activities), and (2) RRs were determined for studies that classified PI using the WHO guidelines [5]. If the meta-analysis did not report the RR stratified by sex or age, we used the same values for both sexes and age-groups. In cases where meta-analysis results compared more than two strata of PI, we combined the natural logs of the RR by weighting component values by the inverse of their variance to generate RR comparing the PA (≥ 600 MET-minute per week) to PI (< 600 MET-minute per week) categories. Ultimately, the RR we use for incident T2D (RR = 0.78) and stroke (RR = 0.80) in PA compared to PI adults were obtained from a meta-analysis published by the Global Burden of Disease group [8]. The RR for the association between PA and CHD (0.86 for men, 0.76 for women) were obtained from the Sattelmair et al. meta-analysis [7].

### Simulations and assumptions

We used the CVD Policy Model-Mexico to simulate two scenarios. First, we simulated the impact of the increase in PI from 2006 to 2012 among adults 35–64 years of age on incident T2D, CHD and stroke and CHD, stroke and MI mortality. We compared outcomes after running separate 7-year simulations, one using PI prevalence from the 2006 ENSANUT and the second using inputs from the 2012 survey. The difference in cumulative disease outcomes represents the impact on disease burden over a 7-year period associated with increased PI observed by 10-year age categories and sex in 2012. For the second scenario, we estimated the impact of a 10% relative reduction in the prevalence of PI between 2016 to 2025 and 5% relative reduction in the prevalence of PI between 2026 to 2030, in accordance with the World Health Organization targets [9]. We used the prevalence of PI observed in 2012 as the starting prevalence in 2016 given findings of no statistical evidence of a difference in PI between those years [31]. We modeled gradual linear decreases in PI over time among 35–64 year-old adults to achieve the overall 10% by 2025 and 15% by 2030. We estimated the probability of having a CV event by stratifying the levels of PI into various risk factors (tobacco smoke, diastolic and systolic blood pressure, low and high-density cholesterol, diabetes, and body mass index). We maintained this risk factor constant to study the relationship between PI and health outcomes (Additional file 1: Appendix S3).

### Calibration

The CVD Policy Model Mexico was calibrated to national data on the number of stroke and CHD events and the CVD and chronic CHD deaths. We used the population at risk for the event and mortality rates as the denominator. Because the size of the population changed through the years (higher post acute myocardial infarction event rates and mortality) we used the population at risk at the beginning of the year (2010) adjusted by the iterative update population-at-risk estimates from prior rates simulations. Iterations were terminated when all 2010 model events and deaths came within 1% of total events and deaths observed in national data by age and sex (Additional file 1: Appendix S2).

### Sensitivity analysis

Self-report instruments underestimate PI levels when compared with accelerometer data [32]. This underestimation can be adjusted using accelerometer-based equations [6, 33]. In 2012, Hallal et al. published global PI prevalences adjusted for accelerometer estimates [33]. In 2013, a similar adjustment equation derived from accelerometer data was developed for Mexican population to estimate PI prevalence [34]. Based on the Mexican adjusted prevalence, we performed a one-way sensitivity analysis to estimate the effect of the adjusted prevalence on the incidence and mortality of CVD and T2D. We used the same modeling procedure as described above, using accelerometer-adjusted PI prevalences. We compared the differences on health outcomes between unadjusted and adjusted PI prevalences. We used Monte Carlo simulations, written in Phython, to estimate the uncertainty of unadjusted and adjusted 7-year period projections and for the gradual linear decrease in PI from 2016 to 2025 and 2030 on the incidence and mortality of CVDs and T2D. In total, 1000 random draws from a standard normal distribution were generated for each health outcome. The SE for each health outcome were calculated using SPSS software version 25 (IBM SPSS statistics, IBM Corporation, Somers, NY).

## Results

The prevalence of PI increased from 2006 to 2012 among all age groups (Table 2), with the highest prevalence in 55–64-year olds (14.4% for 2006 and 16.6% for 2012) and lowest prevalence in 35–44-year olds.

**Table 2:** Prevalence of physical inactivity among Mexican adults: Mexican National Health and Nutrition Survey (ENSANUT) 2006 and 2012

Age-group (years)	2006 (base-case scenario)			2012 (estimates with the increment)			% change from 2006 to 2012
	N (survey)	N (population)	% (95% CI)	N (survey)	N (population)	% (95% CI)	
35–44	11,287	13,542,328	10.8 (9.8, 11.9)	2783	15,843,461	16.3 (13.7, 19.2)	51
45–54	7114	9820,085	12.3 (11.0, 13.6)	1995	11,475,090	14.9 (12.4, 17.8)	21
55–64	4594	6484,080	14.4 (12.8, 16.1)	1495	8416,371	16.6 (13.5, 20.1)	15
Total	22,995	29,846,493	12.1 (11.3, 12.9)	6273	35,734,922	15.9 (14.2, 17.7)	31

Over a 7-year period (from 2006 to 2012), the simulated estimates based on the unadjusted 2012 PI prevalence suggested that there were 27,100 ± 155.1 more cases of T2D, 10,300 ± 99.5 more cases of CHD, 2200 ± 19.2 more cases of stroke, 1500 ± 17.5 more cases of MI, 350 ± 4.1 more deaths from CHD and 400 ± 3.7 more deaths from stroke compared to simulation estimates based on the 2006 PI prevalence (base-case scenario). These additional cases represented relative increases of 1%, 0.8%, 0.8%, 0.5%, 0.2% and 0.6% respectively (Table 3). Sensitivity analyses using the self-reported PI prevalences adjusted using the accelerometer equations PI generated reductions in disease burden that were approximately 50% higher than what was projected using unadjusted self-reported PI prevalence (Table 3).

**Table 3:** Projected accumulated number of cases based on a 7-year period among 35–64 years old Mexicans under different assumptions based on unadjusted and adjusted 2006 physical inactivity prevalence and 2012 physical inactivity prevalence

Outcome	ENSANUT 2006^a^	ENSANUT 2012^b^			
	Base case scenario	Estimates based on unadjusted prevalence of physical inactivity		Estimates based on adjusted prevalence of physical inactivity	
		Increase in events	Percent difference change	Increase in events	Percent difference change
	N	N	%	N	%
Incidence of CHD	1267,400 ± 9.7	10,300 ± 99.5	0.8	14,200 ± 122.1	1.1
Incidence of stroke	293,400 ± 2.6	2200 ± 19.2	0.8	3200 ± 21.8	1.1
Incidence of T2D	2586,300 ± 26.4	27,100 ± 155.1	1.0	34,000 ± 164	1.3
Total myocardial infarction	294,000 ± 2.8	1500 ± 17.5	0.5	2300 ± 23.5	0.8
CHD mortality	141,900 ± 0.6	350 ± 4.1	0.2	600 ± 5.5	0.4
Stroke mortality	67,000 ± 0.5	400 ± 3.7	0.6	600 ± 4.2	0.9

Plus-minus values—means and SE from the Monte Carlo simulations

Values were rounded to the nearest 100 for all outcomes

Unadjusted—self reported physical inactivity

Adjusted—self reported physical inactivity adjusted for accelerometer values

*CHD* coronary heart disease, *T2D* type 2 diabetes

^a^Estimates are based on physical inactivity prevalence in 2006 (base case scenario)

^b^Estimates are based on physical inactivity prevalence in 2012

**Fig. 1:**
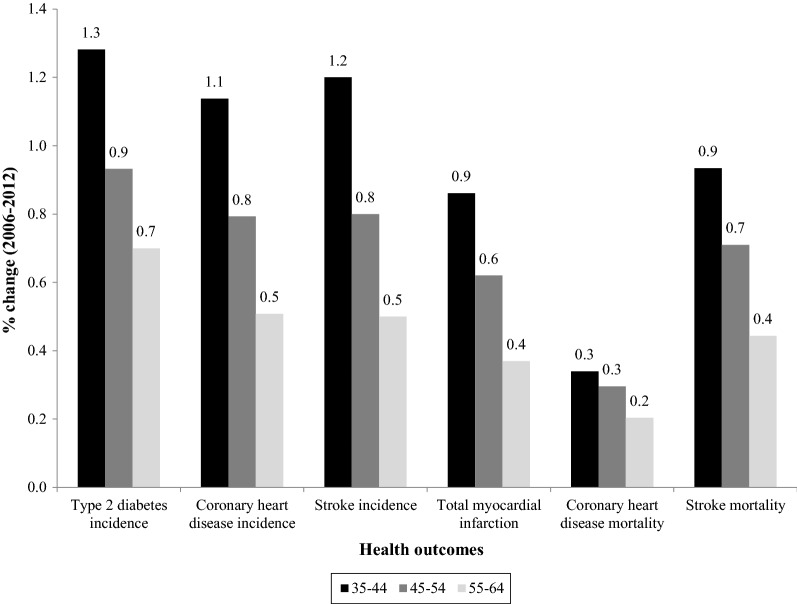
Estimated percentage increase in the number of CVD cases among Mexican adults, according age groups, that were attributable to the increase in the unadjusted prevalence of physical inactivity from 2006 to 2012

The modeled relative increase in events and deaths associated with the higher PI in 2012 compared to 2006 was highest among the youngest (35–44 years) age group and smallest among the oldest (55–64 years) age group (Fig. 1). The estimated increase in cases in the different age groups ranged from 2400 to 14,500 for T2D, 2100 to 4500 for CHD, 620 to 1000 for stroke, 200 to 600 for MI, 50 to 200 for CHD deaths, and 120 to 180 for stroke deaths.

**Fig. 2:**
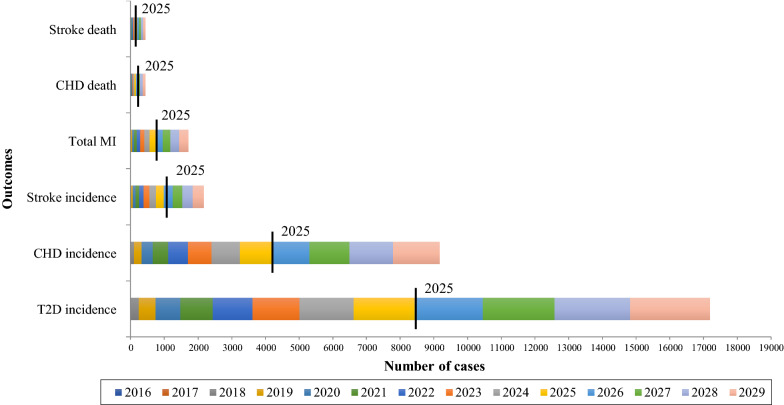
Predicted incident cases of T2D, MI, non-fatal and fatal CHD and stroke among 35–64-year-old Mexican adults that would be prevented from 2016 to 2030 period if there was a 10% and 15% reduction in the prevalence of PI

Simulations that were based on a hypothetical 10% relative decrease in the prevalence of PI from 2016 to 2025 compared to assuming no change in PI resulted in an accumulated decrease of 8400 (0.2%) cases of T2D, 4200 (0.2%) cases of CHD, 1000 (0.2%), cases of stroke, 700 cases of MI (0.1%), 200 deaths of CHD (0.1%) and 200 deaths of stroke (0.2%). Estimates suggested that a 15% reduction in PI from 2026 to 2030 would result in even larger decreases in the number of cases of these diseases (8800 for T2D, 5000 for CHD, 1200 for stroke, 1000 for total MI, 300 for CHD mortality and 200 for stroke mortality) (Fig. 2). Using accelerometer-adjusted prevalences, the hypothetical impact of a 15% reduction on PI from 2016 to 2030 was slightly higher for all health outcomes (19% T2D, 19% CHD, 18% stroke, 18% MI, 18% CHD mortality and 17% stroke mortality).

## Discussion

We projected that the increase in PI prevalence among 35–64-year-old Mexicans from 12.1% in 2006 to 15.9% in 2012 resulted in increases in the incidence and mortality of T2D and CVD (1%: T2D, 0.8%: CHD and stroke incidence, 0.5%: MI, 0.2%: CHD mortality, 0.6%: stroke mortality). By contrast, our estimates suggested that a hypothetical 10% relative decrease in PI prevalence from 2016 to 2025 would result in 0.2% fewer cases of T2D, CHD and stroke, 0.1% fewer cases of MI, 0.1% fewer deaths of CHD and 0.2% fewer deaths of stroke. It is likely that even more events and deaths would be prevented if the WHO target of a 15% relative reduction in PI by 2030 were achieved.

The ENSANUT 2006 and 2012 used the Spanish version of the short form IPAQ to estimate the prevalence of PI. This instrument has been validated in several setting and countries, including Mexico demonstrating an underestimation of PI values [19, 20]. For this reason, this study tried to correct this underestimation by using the PI prevalence adjusted by accelerometer through a sensitivity analysis [6]. Results indicated that an increment of almost 50% on the projected number of cases could be observed when the prevalence is adjusted. Although an adjusted value could be an improved estimation over using the unadjusted prevalence, we consider accelerometer-base prevalence should be used to obtain the closest effect on health of PI.

The WHO has called for a 10% relative reduction in the prevalence of PI by 2025 and a 15% relative reduction by 2030 [9]. Our analysis suggested that a hypothetical 10% relative reduction in PI prevalence by 2025 would result in 0.1 to 0.2% fewer cases of T2D, CHD, stroke, MI and stroke and CHD deaths. The corresponding values for a 15% decrease in PI prevalence by 2030 were 0.2% to 0.6%, respectively. To our knowledge, three previous studies estimated the extent to which a reduction in the prevalence of PI would influence morbidity and mortality. The first of these studies, which was based on the 2008 Australian population, estimated that an absolute 10% reduction in PI prevalence (from 70 to 60%) would decrease the number of deaths by 15% and the loss of disease adjusted life years by 14% [35]. The second of these studies, which is based on the 2002 Colombia population, estimated that a 30% reduction in PI prevalence (from 53.2 to 37.2%) would decrease deaths attributed to T2D by 5.3%, and all-cause mortality by 2% [10]. The final study, which is based on the 1995 Canadian population, estimated that a 10% relative reduction in PI levels (from 62 to 55.8%) would result in a 19.9% fewer T2D deaths, 19.9% fewer stroke deaths, and 10.3% fewer deaths from all-causes [11].

Two of the main reasons for the differences in the estimated decrease in NCD burden in Mexico and these countries is the differences in the targeted reduction in PI prevalence, which ranged from 10 to 30% on a relative scale [10, 11, 35] and differences in the baseline PI prevalences, which ranged from 53.2 to 70% [10, 11, 35]. There were also differences in the prevalence and incidence of NCDs across countries, the age-range of the population the estimates were generated for (from as young as15 to as old as 64 years), the length of time proposed to reduce PI levels, and the RR used in the simulation models. [10, 11, 35] Although, the results are not directly comparable given these many methodological differences, the current study and the three previous studies provide a valuable contribution to the literature regarding the burden of PI and the potential benefit that reducing PI on NCD burden.

The WHO has developed a global action plan to help countries scale up policy actions to reduce PI [9]. The main objectives include creating active societies, creating active environments, creating active people and creating active systems [9]. Although Mexico has implemented some local and federal strategies that may directly or indirectly change PA levels within the population, people is still becoming less active [9]. This study projected that the incidence and mortality of CVD and T2D would likely be reduced if Mexico was able to successfully decrease the prevalence of PI in the coming years. In order to be successful at doing so, local and federal governments will need to work in collaboration with many other sectors (e.g.: health, urbanism, transportation) to identify, adopt and implement cost-effective strategies that could encourage people to be more PA, especially if the country is expected to achieve a 15% reduction of PI prevalence by 2030.

The main strengths of this study are the estimation of the number of cases and deaths based on the new WHO PA goals, the use of a well-established CVD model adapted to the Mexican population, the use of cross-national representative surveys, and the stratification by potential risk factors (e.g.: systolic blood pressure, LDL and HDL, smoking status, body mass index and diabetes) [36]. However, this study has several limitations. The absence of the current prevalence estimates for T2D, hypertension and obesity. We assumed that the prevalence of these risk factors was constant over the years, and this could result in an underestimation of the effect. This assumption was based on the fact that the national prevalence of obesity [37], T2D [38] and hypertension [39] did not significantly changed from 2006 to 2012. However, caution should be taken specifically in the 2016–2025 and 2026–2030 projections, because these estimates could change if some preventive strategies for CVDs change the risk factors prevalence in the near future.

Another potential limitation is that RRs of the association between PI and NCDs used to populate the CVD Policy Model may underestimated or overestimated the projected estimates [7, 8]. First, RRs were obtained from meta-analysis of different prospective cohort studies, which may not represent the real benefit compared to randomized control trials. Second, in some cases, where RRs were not stratified by sex and/or age-groups, the same RR was used for the entire sample. This may result in underestimation or overestimation of the projected estimates in some of the age groups. Third, the RRs were primarily from studies of European, North American and/or Asian countries, which may not represent the true association within Latino populations. Fourth, RRs were mostly obtained from self-reported PA, which may have caused an underestimation of the RRs and subsequently and underestimation of the modeled estimates derived in this study.

## Conclusion

Based on simulation-model estimates, we observed that the rise on the prevalence of PI in 35–64-year-old Mexicans between 2006 and 2012 could have contributed to 27,100 cases of T2D, 10,300 cases of CHD, 2200 cases of stroke, 1500 cases of MI, 350 deaths of CHD and 400 deaths of stroke. In the hypothetical scenario that Mexico may reduce 10% the prevalence of PI by 2025, 8400 cases of T2D, 4200 cases of CHD, 1000 cases of stroke, 700 cases of MI, 200 deaths of CHD and 200 deaths of stroke could be averted. A projected reduction on the PI prevalence of 15% by 2030 could avert 17,100 ± 351.3 cases of T2D, 9200 ± 76.2 cases of CHD, 2200 ± 11.5 cases of stroke, 1700 ± 14.6 cases of MI, 400 ± 3.6 deaths of CHD and 400 ± 2.2 deaths of stroke. Although health benefits estimated from the reduction of PI by 2025 and 2030 are modest, achieving these PI goals would be an important component of the pack of actions that will allow us to move forward in the control of NCDs and T2D in the country and worldwide.

## Supplementary information

**Additional file 1.** Supplementary information for the Cardiovascular Disease Policy Model – Mexico (structure, calibration, inputs and framework).

## Data Availability

Inputs used for the CVD Policy Model-Mexico are publicly available. The CVD Policy Model-Mexico could be used per request from the University of California San Francisco. Please contact joanne.penko@ucsf.edu for further information.

## Abbreviations

CI: Confidence interval
CHD: Coronary heart disease
CVD: Cardiovascular disease
CVD Policy Model: Cardiovascular disease policy model
ENSANUT: National Mexican Health and Nutrition Survey
IPAQ: International Physical Activity Questionnaire
MI: Myocardial infarction
NCDs: Non-communicable diseases
PA: Physical activity
PI: Physical inactivity
RR: Relative risk
SE: Standard error
T2D: Type 2 diabetes
US: United States
WHO: World Health Organization
