# Supplementary material for: Cardiovascular and diabetes burden attributable to physical inactivity in Mexico
Source: Cardiovasc Diabetol. 2020 Jun 29;19:99. doi: 10.1186/s12933-020-01050-3 (PMC7325101; doi:10.1186/s12933-020-01050-3)
Supplement: Supplementary file 1 — Additional file 1. Supplementary information for the Cardiovascular Disease Policy Model – Mexico (structure, calibration, inputs and framework). [file 12933_2020_1050_MOESM1_ESM.docx]

**Additional file 1. Supplementary information for the Cardiovascular Disease Policy Model – Mexico (structure, calibration, inputs and framework)**

**Appendix 1. Cardiovascular Disease Policy Model – Mexico**

**
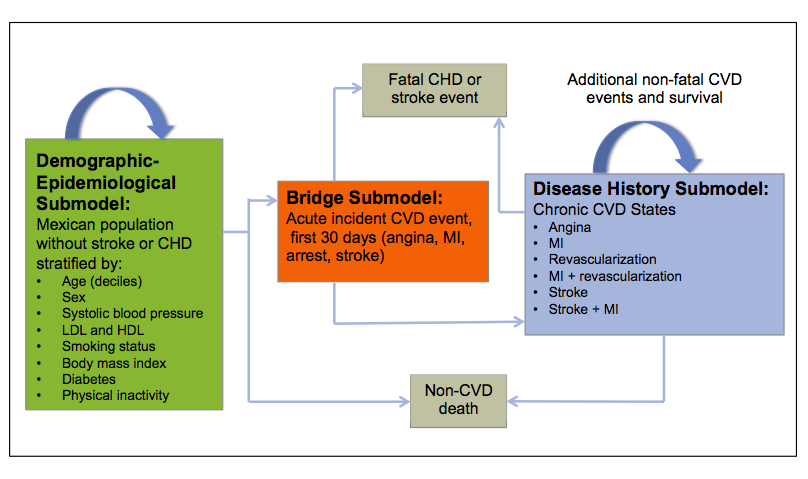
**

**Appendix 2. Comparison between national incidences of CVD and CHD deaths calculated with the Cardiovascular Disease Policy Model. Mexico, 2010**

**Appendix 3. Inputs used for the Cardiovascular Disease Policy Model – Mexico**

**Population**

Adults’ population estimates (35 to 94 years old) were obtained from the National Institute of Statistics, Geography and Informatics (by its acronym in Spanish: INEGI).[1] The population projection was obtained by the Consejo Nacional de Población (CONAPO).[2]

**Risk factors**

The prevalence of the seven risk factors (tobacco smoke, diastolic and systolic blood pressure, low and high-density cholesterol, diabetes, and body mass index) and the correlations among risk factors were obtained from the Smoking Adult Survey 2009[3] and the Mexican National Health and Nutrition Survey (ENSANUT) 2006.[4]

**CVD incidence**

CVD incidence (angina, myocardial infarct, cardiac arrest, ischemic and hemorrhagic stroke) were determined from the 2010 hospitalization electronic databases (Mexican Social Security Institute “Instituto Mexicano del Seguro Social” (IMSS)),[5] The State’s Employees Social Security and Services Institute “Instituto de Seguridad y Servicios Sociales de los Trabajadores del Estado” (ISSSTE),[6] and the Ministry of Health “Secretaría de Salud” (SSA) via the Sistema Nacional de Información en Salud (SINAIS).[7, 8]

**National in-hospital events and CVD deaths**

In-hospital events and deaths were obtained from IMSS, ISSSTE, SSA, the Mexican Petroleum “Petroleos Mexicanos” (PEMEX), Army “Defensa Nacional”, Navy “Marina”, and other public and private hospital systems.[8] CHD deaths from Sistema Nacional de Información en Salud (SINAIS) 2010 general mortality data that includes information. Mortality data was stratified by age, sex, International Classification of Diseases (ICD) 10 codes, and place of death. CHD deaths were estimated using the ICD10 codes (coronary heart diseases: I20-I25, I46 + 2/3 * (I49, I50-I51), and stroke: I60-I69.[9] Non-CVD deaths were considered the difference between CVD deaths (coronary heart disease + stroke) minus the total mortality reported in vital statistics (SINAIS).[10]

**Cardiac arrest (sudden death)**

The total number of cardiac arrests in the population was obtained from the difference between the total CHD deaths (ICD10 codes I20-I25) from the national vital mortality statistics that occurred in hospital settings minus the total of in-hospital deaths (ICD10 codes I20-I25). The result was added to the total number of hospitalizations with an ICD10 code I46 diagnosis from the in-hospital discharge data.

**Arrest survival to hospital**

Information regarding survival to arrest was calculated as the total hospitalizations diagnosed with I46 (ICD10 code) divided by the estimated total arrest.

**Revascularization rates**

The revascularization rate from the USA National Hospital Discharge Survey 2000 (NHDS) was adjusted to reflect a repeat revascularization rate of 2% for percutaneous transluminal coronary angioplasty (PTCA) and 2% for coronary artery bypass grafting (CABG) within the first year. A trend in the ratio of PTCA to CABG was estimated for 2000-2004. PTCA was included as part of the treatment for MI in the same proportion observed in the NHD dataset for 2000, with emergency CABG complicating 2% of these procedures. We included reductions in mortality and re-MI rates for patients treated with PTCA.[11, 12]

**Risk functions for incident T2D, CHD and non-CVD death**

Incident T2D, CHD cases (MI, arrest, or angina) and non-CHD deaths in each risk factor cell for the at-risk population were determined by risk functions $r$ incorporating age sex-specific parameters $\alpha$ and risk factor specific betas, $\left\{\beta_{k}\right.,k=1,2,3,...,6\}$, which are constant over the time span of a simulation, a cell-specific risk factor means, $\left\{m_{k},k=1,2,3,...,6\}\right.$, which are altered by user-defined intervention. The risk function is defined as:

$$r=e^{(\alpha+\sum_{k=1}^{6} \beta_{k}m_{k})}/(1+e^{(\alpha+\sum_{k=1}^{6} \beta_{k}m_{k})})$$

Waves 1-7 from the Framingham Offspring Cohort and 13-28 from the Framingham Heart Study Cohort were used to calculate the risk function beta coefficients for non-CVD deaths, incident CHD events, incident stroke events and type II diabetes events.[13-15] Multivariate model for CHD incident included systolic blood pressure, LDL, smoking, HDL, and diabetes. For stroke and non-CVD death models were adjusted for systolic blood pressure, smoking and diabetes. For type II diabetes the model was adjusted for BMI and age.[16]

CHD, T2D and stroke incidence and non-CVD deaths were stratified by age and sex for 2010 by adjusting the Framingham incidence estimates to account for the differences in risk factor distribution compared with Mexican population as measured in 2006 ENSANUT. The corresponding values of the intercepts by iterative fitting of the risk function to the overall incidence were estimated. It was assumed all risks and rates to be constant over time, since there is no a trend evidence. The CHD, stroke, T2D and non-CVD deaths risk function are applied to every state in every year of a simulation to accommodate the competing risk for these outcomes naturally over time.

It was assumed that all the risk factors modify the incidence of angina, infarction and arrest equally except for smoking. It was assumed that smokers had a higher risk (1.26 relative risk) for arrest and myocardial infarction and a lower excess risk for angina.[17, 18] The Framingham predictor scores have been used in multiple studies for all over the world.[13, 19, 20]

**The transition between risk factors**

Transfers from one risk factor level to another were included to preserve the 2006 ENSANUT age- and gender-specific proportions of the population with each risk factor level.[21] The annual transfer rate was calculated to reduce low-risk population, without regard to the reason of the change, but taking into account the competing risk effect of the Model’s CHD incidence and non-CVD death rates of the base case.

**Appendix 4. Framework for the impact of physical inactivity on health outcomes**

For the model, we assumed the physical inactivity has direct effects on model outcomes including T2D, CHD and stroke. Risk functions defining the relationship between physical inactivity and health outcomes were based on published literature.


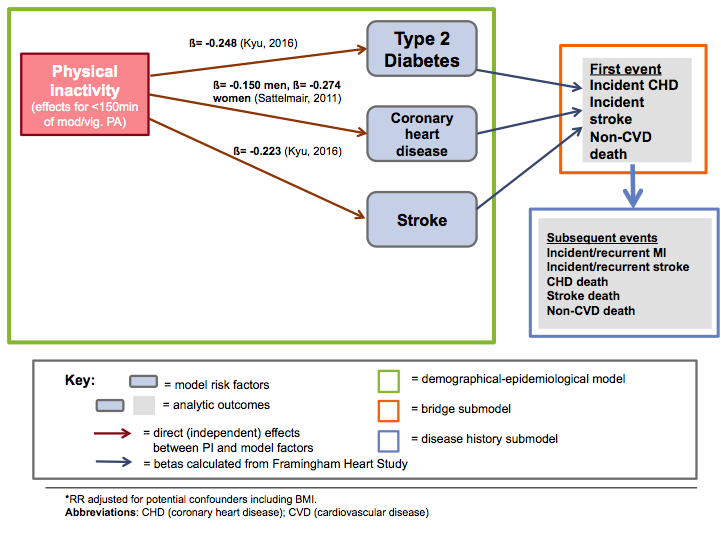


**References**

1. **Estadística población, hogares y vivienda. Instituto Nacional de Estadística, Geografía e Informática.** [<https://www.inegi.org.mx>]. 20 January 2020

2. **Proyecciones de la población 2010-2050. Secretaría de Gobernación.** [<https://www.gob.mx/conapo/acciones-y-programas/conciliacion-demografica-de-mexico-1950-2015-y-proyecciones-de-la-poblacion-de-mexico-y-de-las-entidades-federativas-2016-2050>]. 20 January 2020

3. Reynales Shigematsu L, Shamah T., Méndez I., Rojas R., Lazcano E.: **Encuesta Global de Tabaquismo en Adultos (GATS)**. Cuernavaca, Morelos, México: Organización Panamericana de la Salud e Instiuto Nacional de Salud Pública; 2010.

4. Olaiz-Fernández G, Rivera-Dommarco J., Shamah-Levy T., Rojas R,Villalpando-Hernández S., Hernández-Avila M., Sepúlveda-Amor J.: **Encuesta Nacional de Salud y Nutrición 2006**. Cuernavaca, México: Instituto Nacional de Salud Pública; 2006.

5. **Memoria estadística 2011. Instituto Mexicano del Seguro Social.** [<http://www.imss.gob.mx/conoce-al-imss/memoria-estadistica-2011>]. 20 January 2020

6. **Estadística: anuarios 2011. Instituto de Seguridad y Servicios Sociales de los Trabajadores del Estado.** [<http://www.issste.gob.mx/datosabiertos/anuarios/anuarios2011.html>]. 20 January 2020

7. **Secretaria de Salud. Observatorio del desempeño hospitalario 2011**. In*.* México: Dirección General de Evaluación del Desempeño.; 2012.

8. **Bases de Sector Salud. Cubos Dinámicos-Egresos Hospitalarios 2012. Sistema Nacional de Información de Salud. Dirección General de Información en Salud. Secretaría de Salud.** [<http://www.dgis.salud.gob.mx/contenidos/basesdedatos/bdc_egresoshosp_gobmx.html>].

9. **World Health Organization. ICD-10 Version: 2010. World Health Organization.** [<http://apps.who.int/classifications/icd10/browse/2010/en>].

10. **Bases de Sector Salud. Cubos Dinámicos-Egresos Hospitalarios 2012. Sistema Nacional de Información en Salud. Dirección General de Información en Salud. Secretaría de Salud.** [<http://www.dgis.salud.gob.mx/contenidos/basesdedatos/bdc_egresoshosp_gobmx.html>]. 20 January 2020

11. Lagerqvist B, Husted S, Kontny F, Naslund U, Stahle E, Swahn E, Wallentin L: **A long-term perspective on the protective effects of an early invasive strategy in unstable coronary artery disease: two-year follow-up of the FRISC-II invasive study**. *Journal of the American College of Cardiology* 2002, **40**(11):1902-1914.

12. Zijlstra F, Hoorntje JC, de Boer MJ, Reiffers S, Miedema K, Ottervanger JP, van 't Hof AW, Suryapranata H: **Long-term benefit of primary angioplasty as compared with thrombolytic therapy for acute myocardial infarction**. *The New England journal of medicine* 1999, **341**(19):1413-1419.

13. Brindle P, Emberson J, Lampe F, Walker M, Whincup P, Fahey T, Ebrahim S: **Predictive accuracy of the Framingham coronary risk score in British men: prospective cohort study**. *BMJ* 2003, **327**(7426):1267.

14. D'Agostino RB, Sr., Grundy S, Sullivan LM, Wilson P: **Validation of the Framingham coronary heart disease prediction scores: results of a multiple ethnic groups investigation**. *Jama* 2001, **286**(2):180-187.

15. Liu J, Hong Y, D'Agostino RB, Sr., Wu Z, Wang W, Sun J, Wilson PW, Kannel WB, Zhao D: **Predictive value for the Chinese population of the Framingham CHD risk assessment tool compared with the Chinese Multi-Provincial Cohort Study**. *Jama* 2004, **291**(21):2591-2599.

16. Biggs ML, Mukamal KJ, Luchsinger JA, Ix JH, Carnethon MR, Newman AB, de Boer IH, Strotmeyer ES, Mozaffarian D, Siscovick DS: **Association between adiposity in midlife and older age and risk of diabetes in older adults**. *JAMA* 2010, **303**(24):2504-2512.

17. Parish S, Collins R, Peto R, Youngman L, Barton J, Jayne K, Clarke R, Appleby P, Lyon V, Cederholm-Williams S *et al*: **Cigarette smoking, tar yields, and non-fatal myocardial infarction: 14,000 cases and 32,000 controls in the United Kingdom. The International Studies of Infarct Survival (ISIS) Collaborators**. *BMJ* 1995, **311**(7003):471-477.

18. Law MR, Morris JK, Wald NJ: **Environmental tobacco smoke exposure and ischaemic heart disease: an evaluation of the evidence**. *BMJ* 1997, **315**(7114):973-980.

19. D'Agostino RB, Sr., Grundy S, Sullivan LM, Wilson P, Group CHDRP: **Validation of the Framingham coronary heart disease prediction scores: results of a multiple ethnic groups investigation**. *JAMA* 2001, **286**(2):180-187.

20. Liu J, Hong Y, D'Agostino RB, Sr., Wu Z, Wang W, Sun J, Wilson PW, Kannel WB, Zhao D: **Predictive value for the Chinese population of the Framingham CHD risk assessment tool compared with the Chinese Multi-Provincial Cohort Study**. *JAMA* 2004, **291**(21):2591-2599.

21. Olaiz-Fernández G, Rivera-Dommarco J, Shamah-Levy T, Rojas R, Villalpando-Hernández S, Hernández-Avila M, J. Sl-A: **Encuesta Nacional de Salud y Nutrición 2006.** Cuernavaca, México: Instituto Nacional de Salud Pública; 2006.
